# Hydroxyapatite-coated cementless total hip arthroplasty for patients undergoing dialysis: a study of 30 hips with a minimum follow-up period of 5 years

**DOI:** 10.1186/s12891-021-04718-3

**Published:** 2021-09-30

**Authors:** Akira Hashimoto, Motoki Sonohata, Sakumo Kii, Shunsuke Kawano, Masaaki Mawatari

**Affiliations:** 1 Department of Orthopaedic Surgery, Faculty of Medicine, Saga University, Nabeshima 5-1-1, Saga, 849-8501 Japan; 2 Research Center of Arthroplasty, Faculty of Medicine, Saga University, Nabeshima 5-1-1, Saga, 849-8501 Japan

**Keywords:** Cementless, Complication, Dialysis, Mid-term results, Total hip arthroplasty

## Abstract

**Background:**

The number of total hip arthroplasties (THAs) performed for patients undergoing dialysis is increasing. However, there are few reports of cementless THA for patients undergoing dialysis. This study investigated the mid-term to long-term results of hydroxyapatite (HA)-coated cementless THA for dialysis patients.

**Methods:**

This single-center, retrospective study enrolled dialysis patients undergoing primary HA-coated cementless THA. A total of 24 patients (30 hips) were included in the final analyses. The Harris hip score and radiographic results were assessed preoperatively and during the final follow-up examination. Postoperative complications and mortality rates were recorded. The mean follow-up period was 109 months (range, 60–216 months).

**Results:**

The total Harris hip score significantly improved from 40 to 84 points. The overall cumulative survival rates with revision as the endpoint were 100% at 5 years and 90.4% at both 10 and 15 years. Stress shielding was observed in 24 hips (80%). No deaths were related to the primary THA. Complications included periprosthetic fracture for one patient (3.3%), blood transfusion for nine patients (30%), shunt blockage for two patients (6.7%), deep infection for one patient (3.3%), and dislocation for two patients (6.7%).

**Conclusions:**

HA-coated cementless THA resulted in good mid-term outcomes for patients undergoing dialysis with no mortality risk. However, the procedure involved a relatively high perioperative risk of blood transfusion.

**Supplementary Information:**

The online version contains supplementary material available at 10.1186/s12891-021-04718-3.

## Background

The global prevalence of chronic kidney disease was approximately 13% in 2015 [1]. Hypertension, chronic glomerulonephritis, and diabetes mellitus are the most common causes of chronic kidney disease [2]. Some patients with these conditions may require dialysis despite initial medical management [3]. The risk of needing total hip arthroplasty (THA) is increasing for dialysis patients because of aging and β2-microglobulin amyloid deposits, which contribute to joint destruction and osteonecrosis associated with corticosteroids [4]. For patients undergoing dialysis, the relative risk of needing THA is 6.6 [5]. The number of patients undergoing dialysis is increasing worldwide, as is the number of THA procedures performed for dialysis patients [6, 7].

There have been numerous studies of THA for dialysis patients, and they reported increased rates of major complications, such as infection, mortality, loosening, and hip dislocation [8–10]. However, most of these studies assessed cemented THA because orthopedic surgeons are concerned about the difficulty performing the initial press-fitting to obtain bone ingrowth under poor bone stock and maintaining bone ingrowth stability with ongoing osteodystrophy in dialysis patients [11]. There have been few reports of cementless THA for dialysis patients, and these have included only short-term results and/or a small number of cases [11–13]. Knowledge of the mid-term to long-term results are necessary because mortality has consistently decreased for dialysis patients [14]. However, to the best of our knowledge, no published studies have examined cases with a follow-up period more than 5 years after cementless THA for patients undergoing dialysis.

During this study, we investigated the mid-term to long-term results of hydroxyapatite (HA)-coated cementless THA for patients undergoing dialysis.

## Methods

This was a single-center, retrospective study. The study protocol adhered to the ethical guidelines of the 1975 Declaration of Helsinki, and it was approved by the Institutional Review Board of our institution. All patients provided informed consent prior to participation in the study.

A total of 36 hemodialysis patients (43 hips) who had undergone unilateral primary THA at our hospital between August 1999 and November 2014 were initially included. Patients who were followed-up for less than 5 years or had incomplete clinical functional assessments were excluded. Of the initial 36 patients, three (three hips) were lost to follow-up, eight (nine hips) were followed-up for less than 5 years, and one (one hip) had incomplete data. These patients were excluded from the study. Finally, 24 patients (30 hips) who underwent primary THA while on dialysis were enrolled.

All THA procedures were performed by senior surgeons using a posterolateral approach under spinal or general anesthesia, which was chosen at the discretion of the anesthesiologist.

**Fig. 1:**
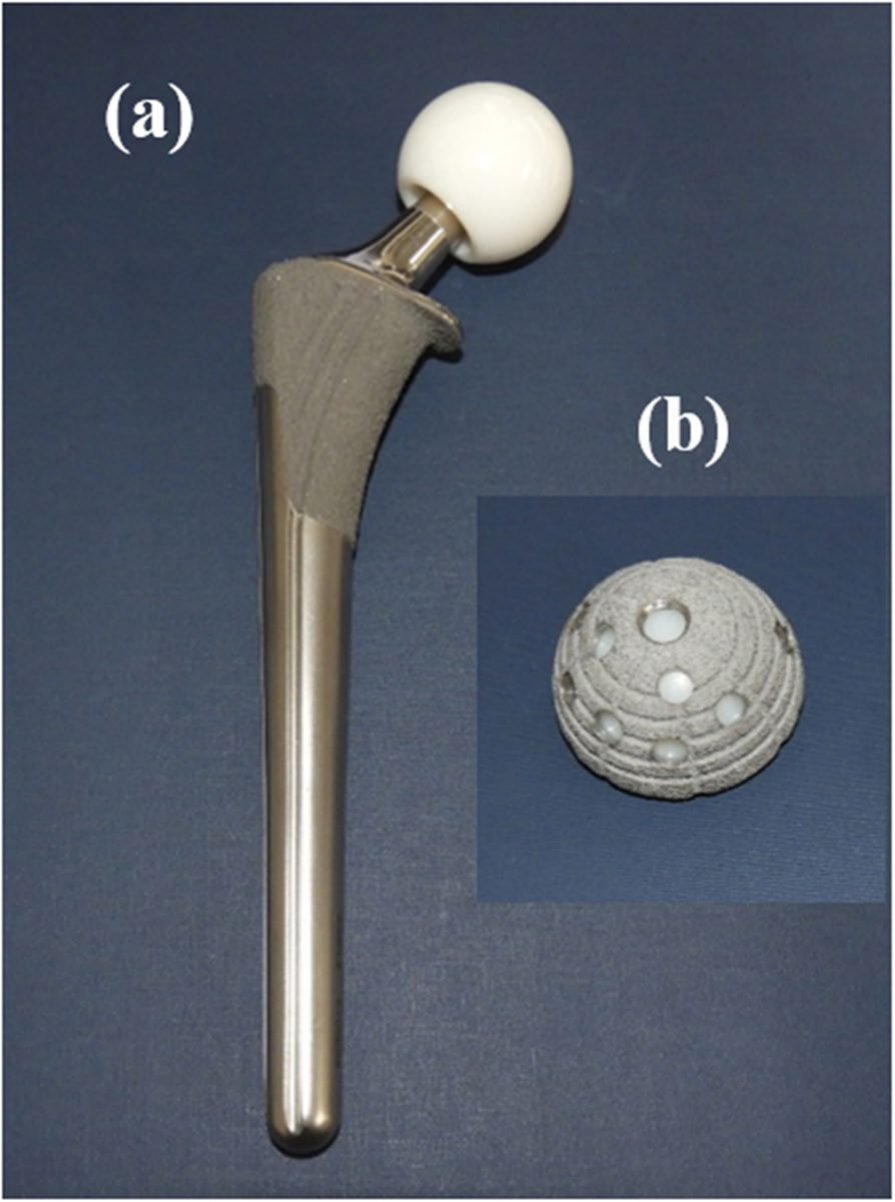
Hydroxyapatite-coated implant. This implant has a porous coating consisting of pure titanium: **a** femoral component and **b** acetabular component

All THA procedures were performed using proximal HA-coated cementless femoral components with a proximal porous coating consisting of pure titanium (PerFix-HA femoral component; Kyocera, Kyoto, Japan) (Fig. 1, Additional file 1), HA-coated cementless hemispherical acetabular shells with a porous coating consisting of pure titanium (AMS-HA acetabular shell; Kyocera, Kyoto, Japan) (Fig. 1, Additional file 1). Initial fixation of the cementless THA component was achieved using the press-fit technique. Two screws were routinely inserted for supplemental acetabular cup fixation, and the additional screws were inserted at the discretion of the surgeons. Autogenous bone grafts using the femoral head were performed for cases of acetabular coverage deficiency at the discretion of the surgeons. The suction drain was removed 2 days postoperatively. To prevent surgical site infection, 1 g cefazolin was administered intravenously before the skin incision and three times within the time period between the patient’s return to the ward and the morning after surgery for all patients. Each patient underwent hemodialysis within 24 h before THA and was not dialyzed again for at least 24 h after surgery. Transfusion was performed at the discretion of the attending physician after considering the postoperative hemodynamics and the degree of anemia on postoperative day 1. Deep venous thrombosis was prevented perioperatively using graduated compression stockings and early walking training without antithrombic drugs for the dialysis group. Walking training within the allowable pain range was started without weight-bearing limitations 2 days postoperatively.

The control group initially included 3584 patients (4194 hips) who did not have a medical history of dialysis and had undergone unilateral primary THA at our hospital between August 1999 and November 2014. Patients who were followed-up for less than 5 years (1284 patients, 1658 hips) or had incomplete clinical functional assessments (200 patients, 214 hips) were excluded. Finally, 2100 patients (2322 hips) who underwent primary THA were enrolled. All THA procedures were performed in the same manner for dialysis patients.

The operative time, intraoperative blood loss, postoperative blood loss, and hemoglobin (Hb) levels on postoperative day 1 were assessed. Intraoperative blood loss was calculated based on the contents of the suction bottle and the change in the weight of the used surgical sponges, as recorded in the anesthetic records. Postoperative blood loss was calculated based on the drain contents.

We used the Harris hip score to perform clinical evaluations preoperatively, at discharge, at 3 months postoperatively, at 6 months postoperatively, at 1 year postoperatively, and approximately every year thereafter (based on the convenience for the patient) [15]. The Harris hip score includes the following four categories: pain, function, deformity, and range of motion.

The preoperative indication for primary THA was assessed using radiographs only or radiographs and magnetic resonance imaging results. Preoperative radiographs were categorized according to the bone stock type using the Dorr cortical bone classification [16]. We performed postoperative radiographic evaluations at 1 week postoperatively, 3 months postoperatively, 6 months postoperatively, 1 year postoperatively, and approximately every year thereafter (based on the convenience for the patient). Postoperative radiographs were examined to assess the fixation status of the cementless components, existence of osteolysis, and degree of stress shielding. Cup loosening was defined as > 5 mm of migration, a change of > 3° in the cup angle, or a complete radiolucent line > 1 mm in the three zones as described by DeLee and Charnley [17, 18]. Stem fixation was evaluated using the criteria described by Engh et al. [19]. Osteolysis was defined as a lytic lesion with a minimal longitudinal measurement of 5 mm that was not observed on the initial postoperative radiograph [12]. The femoral stem was assessed for the presence or absence of osteolysis in the Gruen zones [20]. Stress shielding was assessed according to the criteria described by Engh et al. [21]. The radiographs of patients who did not undergo revision THA were assessed at the latest follow-up, and those of patients who had undergone revision THA were assessed before revision. The radiograph assessment results were evaluated by a senior orthopedic surgeon.

Additional data extracted included mortality, fracture, transfusion, medical complications, infection, dislocation, and sciatic nerve palsy.

Patients were classified into two groups, the revision group and the no revision group, based on the performance of THA revision within the follow-up period.

### Statistical analysis

All numerical data are expressed as the mean ± standard deviation. All analyses were performed using JMP Pro software (version 14.2.0; SAS Institute Japan Ltd., Tokyo, Japan). The Shapiro-Wilk test was conducted to evaluate the distribution normality of the continuous variables. We used the Wilcoxon signed-rank test to compare the Harris hip scores preoperatively and at the latest follow-up examination. The Wilcoxon signed-rank test, Fisher’s exact test, and Pearson chi-squared test were used to compare factors between groups. The Wilcoxon rank sum, Kruskal-Wallis, and Spearman rank correlation tests were used to identify factors affecting intraoperative blood loss. The Wilcoxon rank sum test was used to compare Hb levels of transfused and non-transfused patients on postoperative day 1. The level of significance was set at *p* < 0.05, and survivorship analyses were performed using death and THA revision as endpoints [22].

To minimize confounding, propensity score matching was used to match dialysis patients to non-dialysis patients. Logistic regression using the following seven variables was performed to calculate the propensity score: age, sex, body mass index, preoperative Harris hip score, osteoarthritis, osteonecrosis, and trauma. Then, 1:1 propensity matching was performed using nearest-neighbor matching without replacement, with each dialysis patient was matched to a control non-dialysis patient. A caliper width of 0.2 of the standard deviation of the logit of the propensity score was used. To check the balance of the matches, a standardized mean difference threshold of 0.1 was set as the reference. The Wilcoxon rank sum test or Student t-test was used to compare age, follow-up periods, total Harris hip scores preoperatively, total Harris hip scores at the latest follow-up examination, and perioperative blood loss between the dialysis group and control group. Fisher’s exact test or the Pearson chi-squared test was used to compare sex, reason for THA, Door classification, grade of stress shielding, and presence or absence of transfusion between the dialysis group and control group. The level of significance was set at *p* < 0.05, and survivorship analyses were performed using Kaplan-Meier and log-rank tests with death and THA revision as endpoints [22]. The level of significance was set at *p* < 0.05, and survivorship analyses were performed using death and THA revision as endpoints [22].

## Results

The characteristics of the dialysis group are shown in Table 1. The mean age of patients who underwent primary THA was 55.9 years. The mean follow-up period after primary THA was 108.6 months, and the mean duration of dialysis before primary THA was 144.1 months. Chronic glomerulonephritis was the most common reason for dialysis (14 hips; 46.7%), and osteoarthritis was the most common reason for primary THA (21 hips; 70%).

**Table 1:** Patient characteristics

Hips/patients, n	30/24
Sex, male and female	4 (13.3) and 26 (86.7)
Age at primary THA, years	55.9 ± 9.2 (43–74)
Height, cm	151.0 ± 8.9 (136.8–164.8)
Weight, kg	43.3 ± 7.3 (35.1–59.6)
Body mass index, kg/m^2^	19.5 ± 3.0 (15.7–25.8)
Follow-up period after primary THA, months	111.8 ± 41.0 (60–216)
Causes of renal failure	
Chronic glomerulonephritis	14 (46.7)
Diabetes mellitus	3 (10.0)
Systemic lupus erythematosus	2 (6.7)
Polycystic kidney disease	2 (6.7)
Hypertension	2 (6.7)
Preeclampsia	2 (6.7)
Allergic purpura	2 (6.7)
ANCA-related glomerulonephritis	1 (3.3)
Unknown	2 (6.7)
Duration of dialysis before primary THA, months	144.1 ± 114.4 (2–408)
Preoperative diagnosis for primary THA	
Osteoarthritis	21 (70.0)
Osteonecrosis	8 (26.7)
Fracture	1 (3.3)

Data are presented as n (%) or mean ± standard deviation (range). *THA* total hip arthroplasty, *ANCA* antineutrophil cytoplasmic antibodies

### Clinical results

The total Harris hip score significantly improved from a mean of 40.2 points before THA to 83.7 points at the latest follow-up examination (Table 2). The mean operative time was 39.6 min, the mean intraoperative blood loss was 230.2 g, and the mean postoperative blood loss was 300.8 g (Table 2). A positive correlation was observed between the operative time and intraoperative blood loss (Table 3). No relationship was observed between intraoperative blood loss and any other variable (Table 3). Postoperative red blood cell transfusion was performed for eight patients; these transfusions required and either 2 units (six patients) or 4 units (two patients) (Table 2). The mean Hb level on postoperative day 1 was 9.0 g/dL (±1.8 g/dL). The mean Hb level of transfused patients was 7.9 g/dL (±1.0 g/dL). The mean Hb level of non-transfused patients was 9.4 g/dL (±1.9 g/dL). Therefore, the Hb levels of transfused patients were significantly lower than those of non-transfused patients (*p* = 0.04).

**Table 2:** Clinical results and complications

		*p*-value
Operative time, min	39.6 ± 11.3 (25–57)	
Intraoperative blood loss, g	230.2 ± 109.8 (60–410)	
Postoperative blood loss, g	300.8 ± 209.1 (20–730)	
Harris hip scores		
Total before THA	40.2 ± 11.4 (19–74)	< 0.0001
Total at final follow-up	83.7 ± 11.5 (51–95)	
Pain before THA	13.0 ± 7.0 (0–20)	< 0.0001
Pain at final follow-up	41.1 ± 4.7 (30–44)	
Function before THA	23.6 ± 6.9 (6–30)	< 0.0001
Function at final follow-up	38.4 ± 8.1 (18–47)	
Deformity before THA	0.2 ± 0.6 (0–2)	1.000
Deformity at final follow-up	0.2 ± 0.6 (0–2)	
ROM before THA	3.4 ± 0.8 (2–5)	0.0067
ROM at final follow-up	4.0 ± 0.7 (3–5)	
Autogenous bone graft	13 (43.3)	
Complications		
Fracture	1 (3.3)	
Transfusion	8 (26.7)	
Shunt blockage	2 (6.7)	
Infection	1 (3.3)	
Dislocation	2 (6.7)	
Nerve palsy	0 (0)	
Loosening (acetabular, femoral)	2 (6.7), 1 (3.3)	
Osteolysis (acetabular, femoral)	1 (3.3), 1 (3.3)	

Data are presented as the mean ± standard deviation (range) or number of hips (%). *THA* total hip arthroplasty, *ROM* range of motion

**Table 3:** Correlation between intraoperative blood loss and other variables, including the Dorr classification

Factors	Pearson Correlation Coefficient (ρ)	*p*-value
Continuous variables		
Age at primary THA	−0.1942	0.3039
Height	−0.0493	0.7961
Weight	−0.2672	0.1535
BMI	−0.2789	0.1355
Follow-up period after primary THA	−0.2148	0.2543
Duration of dialysis before primary THA	0.0942	0.6206
Operative time	0.5759	0.0009
Postoperative blood loss	−0.1384	0.4657
Nominal variables		
Sex		0.1999
Cause of renal failure		0.4871
Diagnosis for THA		0.4961
Dorr type		0.1424
Autogenous bone graft		0.0624

*THA* total hip arthroplasty, *BMI* body mass index

### Radiographic results

Regarding the acetabular side, 27 hips (90%) had bone ingrowth fixation. Two hips (6.7%) had aseptic cup loosening requiring THA revision 8 or 15.5 years after primary THA (Tables 2 and 4). Although one hip had osteolysis in zone 2, the patient did not undergo revision because no cup loosening occurred.

**Table 4:** Radiographic evaluation

Acetabular side	
Fixation status at final follow-up	Bone ingrowth fixation (27), Aseptic loosening (2), osteolysis (1)
Femoral side	
Dorr cortical bone classification before primary THA	A (13), B (12), C (5)
Stress shielding at final follow-up	1 (6), 2 (9), 3 (4), 4 (5)
Fixation status at final follow-up	Bone ingrowth fixation (27), fibrous fixation (1), aseptic loosening (1), osteolysis (1)

Numbers in parentheses indicate the number of hips in each category. *THA* total hip arthroplasty

Regarding the femoral side, 27 hips (90%) had bone ingrowth fixation. One hip (3.3%) (Dorr type C before THA) had aseptic loosening; therefore, revision surgery was performed 6.3 years after primary THA (Tables 2 and 4). Although one hip (Dorr type C before THA) had osteolysis in zone 1, the patient did not undergo revision because no stem loosening occurred. Stress shielding was recognized in 24 hips (80%) (Table 4).

### Other complications

Surgical complications are shown in Table 2. Periprosthetic fracture (Vancouver classification AG [23]) occurred in one hip after a fall 18 years postoperatively; that patient was treated conservatively. Postoperative shunt blockage during hospitalization occurred in two hips, and percutaneous transluminal angioplasty was performed for two hips. Deep infection occurred in one hip 4 years postoperatively. Debridement and revision of the ball and liner were performed, and there was no recurrence of infection. Dislocation was observed in two hips within 1 month postoperatively; however, additional surgery was not needed to prevent dislocation. Sciatic nerve palsy was not observed during this study.

### Survival of femoral and acetabular components

**Fig. 2:**
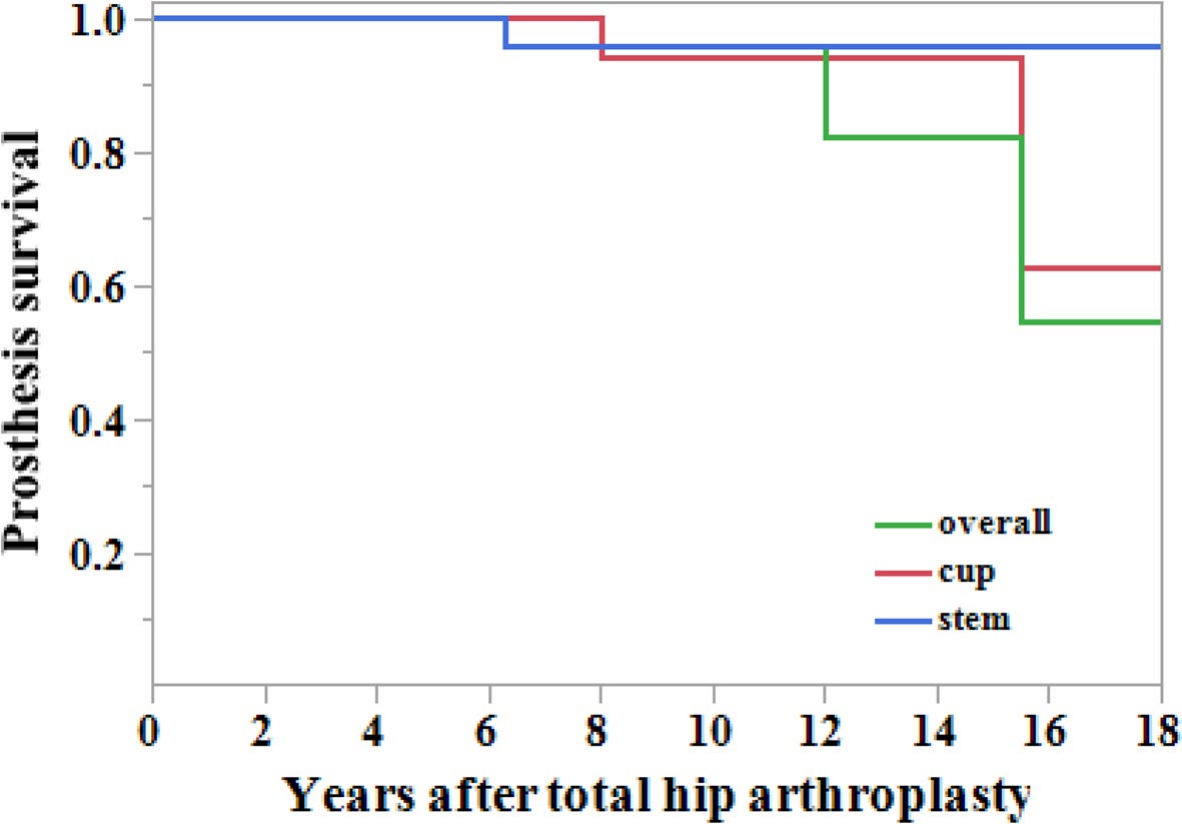
The cumulative survival rate of the implants with revision as the endpoint. The survival rates for the cup were 100% at 5 years, 94.1% at both 10 and 15 years, and 62.8% at 18 years. The survival rates for the stem were 100% at 5 years and 95.8% at 10, 15, and 18 years. The overall survival rates (of both the cup and stem) were 100% at 5 years, 95.8% at 10 years, 82.1% at 15 years, and 54.8% at 18 years

Regarding the cup, the cumulative survival rates with revision as the endpoint were 100% at 5 years, 94.1% at both 10 and 15 years, and 62.8% at 18 years (Fig. 2). Regarding the stem, the cumulative survival rates with revision as the endpoint were 100% at 5 years and 95.8% at 10, 15, and 18 years (Fig. 2). The overall cumulative survival rates with revision as the endpoint were 100% at 5 years, 95.8% at 10 years, 82.1% at 15 years, and 54.8% at 18 years (Fig. 2). No significant differences in all variables were observed between the revision and no revision groups (Table 5).

**Table 5:** Propensity-matched study cohort

Group (hips)	Dialysis group (26)	Control group (26)	*p*-value
Sex, male and female	3 (11.5) and 23 (88.5)	3 (11.5) and 23 (88.5)	1.000
Age at primary THA, years	53.7 ± 8.9 (44–74)	56.2 ± 10.0 (42–79)	0.8907
Height, cm	150.5 ± 8.9 (129.6–165.0)	150.0 ± 8.9 (127.1–157.6)	0.7626
Weight, kg	44.8 ± 7.6 (30.2–59.6)	44.9 ± 5.4 (32.8–56.5)	0.9198
Body mass index, kg/m^2^	19.8 ± 3.1 (15.7–25.8)	20.0 ± 2.1 (16.3–24.7)	0.6018
Follow-up period after primary THA (months)	107.1 ± 42.8 (60–216)	104.8 ± 33.2 (60–168)	0.8290
Preoperative diagnosis for primary THA			
Osteoarthritis	21 (80.8)	22 (84.6)	0.5995
Osteonecrosis	4 (15.4)	4 (15.4)	
Fracture	1 (3.3)	0 (0)	
Operative time, min	40.4 ± 11.7 (25–64)	38.2 ± 9.0 (25–55)	0.4449
Intraoperative blood loss, g	232.2 ± 114.4 (70–410)	243.6 ± 145.1 (50–550)	0.7531
Postoperative blood loss, g	301.3 ± 220.9 (20–730)	438.5 ± 238.0 (120–990)	0.0361
Harris hip scores			
Total before THA	40.5 ± 12.0 (30–74)	39.1 ± 12.0 (18–66)	0.6717
Total at final follow-up	81.3 ± 14.8 (36–95)	87.4 ± 9.2 (62–95)	0.1036
Autogenous bone graft	10 (38.5)	6 (23.1)	0.2294
Transfusion	7 (26.9)	2 (8.7)	0.1400
Radiographic evaluation			
Dorr cortical bone classification before primary THA	A (11), B (11), C (4)	A (6), B (18), C (2)	0.1476
Stress shielding at final follow-up	1 (6), 2 (8), 3 (3), 4 (4)	1 (9), 2 (2), 3 (5), 4 (3)	0.2697

Data are presented as the mean ± standard deviation (range) or number of hips (%). *THA* total hip arthroplasty

### Mortality rate

**Fig. 3:**
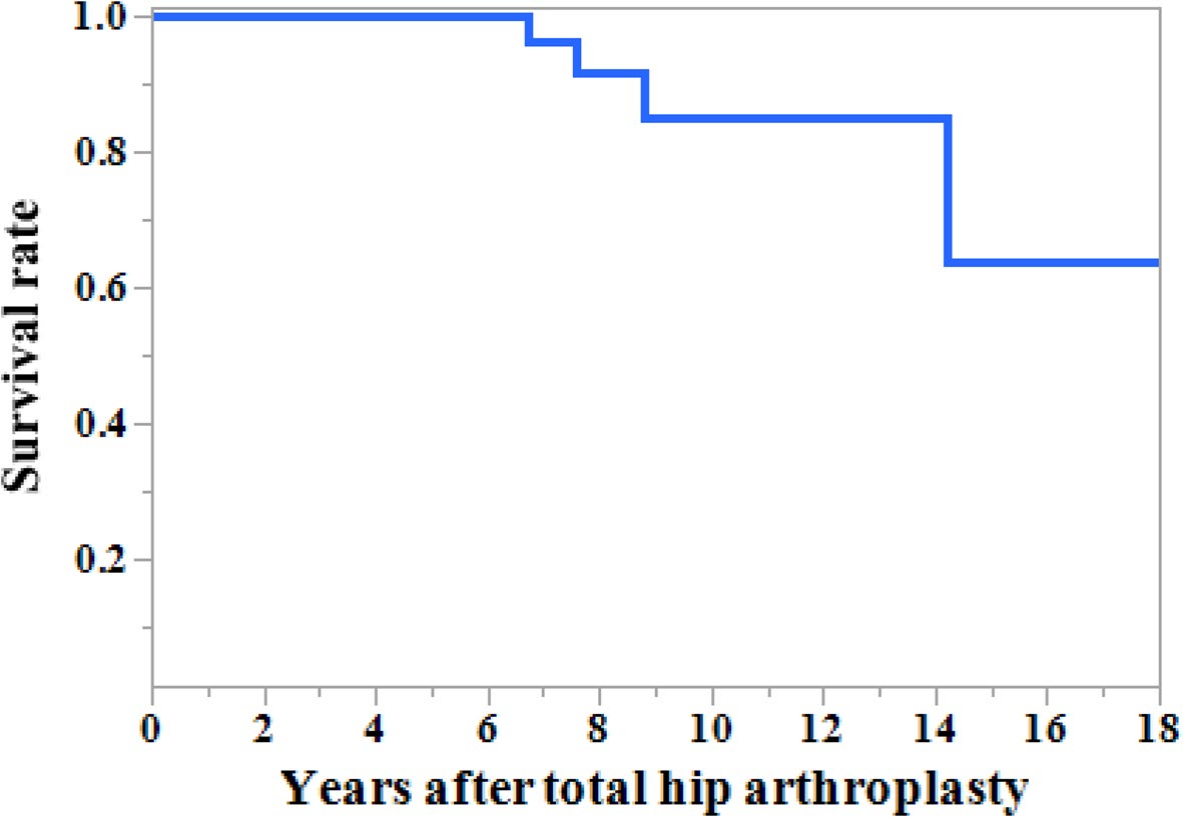
The cumulative survival rate with mortality as the endpoint. The survival rates were 100% at 5 years, 85.0% at 10 years, and 63.7% at both 15 and 18 years

There were no deaths related to the primary THA. Five deaths occurred after primary THA (one case of uremia in an 81-year-old patient at 8.8 years postoperatively; one myocardial infarction in a 76-year-old patient at 14.2 years postoperatively; one cerebral infarction in a 67-year-old patient at 7.6 years postoperatively; one gastrointestinal perforation in a 61-year-old patient at 6.7 years postoperatively; and one abdominal aorta rupture in an 81-year-old patients at 18.8 years postoperatively). The cumulative survival rates with mortality as the endpoint were 100% at 5 years, 85.0% at 10 years, and 63.7% at both 15 and 18 years (Fig. 3).

### Case presentation

**Fig. 4:**
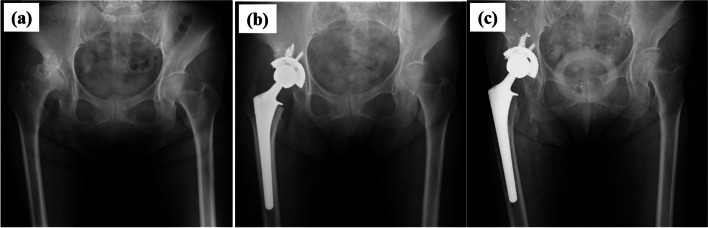
Pelvic radiographs. Radiograph of the pelvis (**a**) before right-side total hip arthroplasty, (**b**) at 2 weeks after right-side total hip arthroplasty, and (**c**) at 68 months after right-side total hip arthroplasty

A 62-year-old woman presented with right hip pain and limited range of motion of the right hip. She had undergone hemodialysis for 68 months at the time of presentation to our hospital. She had a history of hypertension. Her drug, family, and psychosocial histories were irrelevant. Her X-ray examination showed severe osteoarthritis of the right hip (Kellgren-Lawrence grade 4) (Fig. 4-a) [24]. Therefore, she underwent right cementless THA (PerFix-HA femoral component, AMS-HA acetabular shell, AMS liner, zirconia ball; Kyocera, Kyoto, Japan) (Fig. 4-b). No perioperative complications occurred. At 68 months postoperatively, there was bone ingrowth fixation on the acetabular and femoral sides and grade 4 stress shielding on the femoral side (Fig. 4-c) [12, 17–21]. The preoperative total Harris hip score was 54 points; however, it had improved to 87 points by the time of the final observation [15].

### Propensity-matched study cohort

**Fig. 5:**
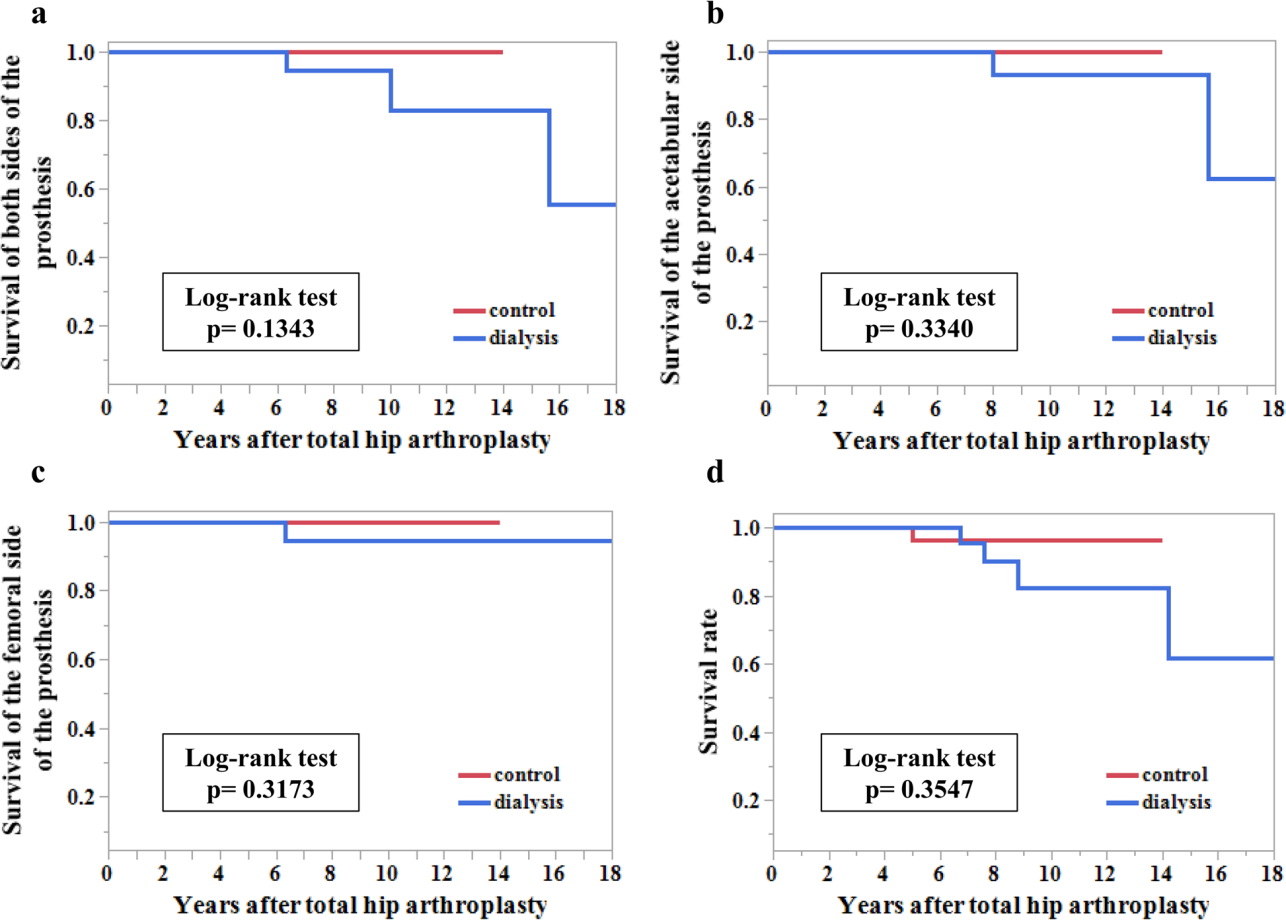
Cumulative survival rates of the dialysis group and the control group. Cumulative survival rates (**a**) of both sides of the prothesis, (**b**) of the acetabular side of the prothesis, (**c**) of the femoral side of prothesis, and (**d**) with mortality

After propensity score matching, 26 hips remained in each group (Table 5, Additional file 2). Patient characteristics, clinical results, complications, and radiographic evaluation results of the control group are shown in Additional file 2. There was no significant difference in all items between the two groups (Table 5). There were no significant differences in the survival of components and mortality rates between the two groups (Fig. 5a-d).

## Discussion

This is the first study to investigate the mid-term to long-term results of primary HA-coated cementless THA for patients undergoing dialysis and to perform follow-up for a minimum period of 5 years. Our findings indicate that primary HA-coated cementless THA results in good mid-term outcomes and is a useful treatment option for dialysis patients.

Cementless THA has not been used for dialysis patients because of concerns regarding poor bone stock, osteodystrophy, and β2-microglobilin deposits, which migrate to the bone-implant interface and lead to implant loosening [11, 25]. However, previous studies reported that the rates of cemented implant loosening were 35% at an average of 5 years after THA and 58% at an average of 7 years after THA [26, 27]. Therefore, cemented THA has not shown promising mid-term results. In contrast, the rate of loosening of cementless implants ranged from 0 to 6% during short-term to mid-term follow-up [11–13]. During our study, the cumulative survival rates for implants with revision as the endpoint were 100% at 5 years, 95.8% at 10 years, and 82.1% at 15 years. Additionally, our clinical results are promising. Therefore, HA-coated implants can lead to good mid-term results of cementless THA for dialysis patients.

HA is a non-toxic, biocompatible, and osteoconductive material [28]. The HA coating accelerates bone healing and enhances the biologic fixation of implants during a short period because of its biocompatibility and osteoconductive ability [29]. Although a previous study reported that the rate of loosening of cemented implants was 35% [27], bone ingrowth fixation was observed in 27 hips (90%) on both the acetabular and femoral sides during our study. We used HA-coated implants during this study; therefore, HA-coated implants may be beneficial for cementless THA for dialysis patients.

During this study, one hip (3.3%) experienced a periprosthetic fracture because of a fall. The risk of fracture increases with longer follow-up periods after THA [30]. Long-term dialysis because of the extended lifespan of dialysis patients may be a risk factor for osteodystrophy, which is a risk factor for fracture [31]. Additionally, poor bone stock caused by stress shielding is a risk factor for periprosthetic fractures [32]. Therefore, longer follow-up periods after THA may show that HA-coated cementless THA may increase the risk of periprosthetic fractures.

One previous study found intraoperative blood loss greater than 500 mL during THA for dialysis patients [33]. Dialysis patients are likely to experience hemorrhage caused by the destruction of plates by the dialysis machine and heparization during dialysis [33]. To avoid heparization, which can lead to bleeding at the surgical site, dialysis within 24 h postoperatively is not recommended [33]. During our study, the mean intraoperative blood loss was 230 g, which is less than that observed during a previous study [33]. Another previous study showed a mean operative time of 65.5 min using the posterolateral approach for primary THA [34]. During our study, the mean operative time was 39.6 min. A long operative time can increase the risks of perioperative blood loss and transfusion during total joint arthroplasty [35]. During this study, a positive correlation was observed between intraoperative blood loss and operative time. Therefore, the relatively low intraoperative blood loss may be attributable to the short operative time [35]. Although the rate of transfusion among primary THA for the general population has been reported to be 16.9% [36], eight patients (24%) required transfusion during this study. Therefore, there was a high risk of transfusion during THA for dialysis patients. Cardiovascular and cerebrovascular events can occur together with hemodynamic instability after perioperative bleeding and transfusion [33]. Hence, it is important to maintain perioperative hemodynamic stability in dialysis patients. During our study, shunt blockage occurred in two patients (6.7%). The cause of shunt blockage may be related to perioperative blood loss. Therefore, expeditious surgery is important to reduce perioperative blood loss and maintain perioperative hemodynamic stability in dialysis patients undergoing THA.

During this study, the overall cumulative survival rates with revision as the endpoint were 82.1 and 54.8% at 15 and 18 years postoperatively, respectively. Hence, considering the long-term results of HA-coated cementless THA, revision THA may be required in the future. The bone economy is an obvious advantage that makes revision THA easier [37]. However, stress shielding was observed in 24 hips (80%) during our study. The insertion of the stem into the intramedullary canal could ​decrease the stress distributed at the bone–implant interface [38]. The distribution of stress around the implanted stem leads to stress shielding, which is a metabolic decrease in bone mass resulting in porous or thin bone [38]. During the past decade, short femoral stems have attracted increasing attention. An advantage of short stems is that less femoral bone stock lock occurs because there is less invasion and less stress shielding on the proximal femur compared to conventional stems [39, 40]. Additionally, the reduction in stress shielding using a short stem may reduce the long-term risk of periprosthetic fracture [41]. The stem design is related to blood loss and the transfusion rate, with less blood loss and lower transfusion rates associated with short stems than with long stems [42]. Although intraoperative blood loss during our study was relatively low compared with that of another study [33], there was a high risk of transfusion to maintain hemodynamic stability. During this study, we used conventional long stems for THA. Hence, the use of short stems may have the potential to decrease perioperative blood loss and reduce the risk of transfusion without the loss of hemodynamic stability. Although short stems have the advantage of less bone stock loss, lower fracture risk, less blood loss, and lower risk of transfusion after THA than long stems [39–42], little is known about the fixation of short stems during THA for dialysis patients. However, HA-coated conventional stems showed promising mid-term results during this study. Additionally, stress shielding had no effect on the survival rate of stems during our study, and stress shielding had no effect on hip function during a previous study [43]. Therefore, the choice of stem design for cementless THA for dialysis patients remains controversial.

A previous study of THA for dialysis patients found a 5.5% mortality rate related to THA, 6.3% all-cause mortality rate at 1 year postoperatively, and a mean time to death of 3.3 years [9]. However, no deaths were related to primary THA during this study. Additionally, our study showed lower mortality rates (cumulative survival rate for mortality: 100% at 5 years; 85.0% at 10 years; and 63.7% at 15 years) and a longer mean time to death (11.2 years) than previous studies. Moreover, the mortality rate of dialysis patients is decreasing [14]. Therefore, studies of THA for dialysis patients should report at least mid-term results, and HA-coated cementless THA, which has shown good mid-term results, may be a useful treatment option for dialysis patients. Chang and Hsieh found a relationship between cardiovascular and cerebrovascular events and hemodynamic instability after perioperative bleeding and transfusion [33]. During our study, lower intraoperative blood loss and high transfusion rates may lead to perioperative hemodynamic stability, resulting in only two shunt blockages and no fatal cardiovascular or cerebrovascular events. Perioperative hemodynamic management to reduce intraoperative blood loss and transfusion may improve the safety of HA-coated cementless THA.

Lieu et al. reported an 8.5% incidence of deep infection after primary THA for dialysis patients [9]. However, other studies found no deep infection after intravenous administration of prophylactic antibiotic for 3 to 5 days after cementless THA for dialysis patients [11, 12]. During this study, intravenous prophylactic antibiotics were administered for 2 days, and only one hip (3.3%) developed a deep infection. Although the cause of infection was unclear, the infection was not acute; furthermore, the infection was delayed and observed at 4 years postoperatively. The prevention of infection with long-term antibiotics is uncommon [44]. Therefore, the antibiotic prophylaxis period to prevent infections remains controversial for dialysis patients undergoing THA.

Dialysis patients are at higher risk for dislocation after THA than patients with no renal disease because their decreased muscular tone and muscle weakness with renal osteodystrophy lead to increased soft tissue laxity [45]. Previous studies have reported a 6.5% rate of dislocation after primary THA for dialysis patients [9, 45]. During our study, dislocation occurred in one hip (6%). Therefore, strict management of the repair or preservation of the soft tissue structure, appropriate component placement, and postoperative protection should be performed to prevent dislocation after THA for dialysis patients [33, 46, 47].

There were four limitations to this study. First, this study included a relatively small sample size and mid-term results. Therefore, future studies involving a larger number of patients and long-term follow-up results are needed. Second, we performed THA with conventional HA-coated cementless stems only; however, other stem designs may produce different results. Hence, a future study of cementless THA using other types of stems should be performed. Third, the presence and absence of the HA coating were not compared. Future studies assessing the presence and absence of the HA coating could provide useful information. Fourth, we investigated femoral bone remodeling and the grade of stress shielding using radiographic evaluations. Evaluations of the bone mineral density around the stem using dual-energy X-ray absorptiometry could provide a precise mathematical assessment, however.

## Conclusion

Primary THA for dialysis patients is associated with increased risks of transfusion, infection, and dislocation compared to primary THA for patients who are not undergoing dialysis. Despite these risks, HA-coated cementless THA resulted in good mid-term outcomes for dialysis patients with no mortality risk. Therefore, HA-coated cementless THA may be a useful treatment option for patients undergoing dialysis.

## Supplementary Information

**Additional file 1.** Details of the implants, specific implants used for each patient, and complications.

**Additional file 2.** Details of the control group.

## Data Availability

The datasets used and/or analyzed during the current study are available from the corresponding author on reasonable request.

## Abbreviations

THA: Total hip arthroplasty
HA: Hydroxyapatite
