# Supplementary material for: Hydroxyapatite-coated cementless total hip arthroplasty for patients undergoing dialysis: a study of 30 hips with a minimum follow-up period of 5 years
Source: BMC Musculoskelet Disord. 2021 Sep 30;22:842. doi: 10.1186/s12891-021-04718-3 (PMC8485528; doi:10.1186/s12891-021-04718-3)
Supplement: Supplementary file 1 — Additional file 1. Details of the implants, specific implants used for each patient, and complications. [file 12891_2021_4718_MOESM1_ESM.docx]

**Additional file 1**

**Detail of the implants**

The PerFix-HA femoral component (Kyocera, Kyoto, Japan) is a proximal hydroxyapatite (HA)-coated cementless femoral component with a proximal porous coating consisting of pure titanium. The AMS-HA acetabular shell (Kyocera, Kyoto, Japan) is an HA-coated cementless hemispherical acetabular shell with a porous coating consisting of pure titanium. An ABS liner (Kyocera, Kyoto, Japan) is a sandwich-type ceramic and alumina-bearing surface liner that is mechanically fixed to a polyethylene liner. Dissociation of the ABS liner has occurred in 649 of 3933 hips (16.5%) according to postmarketing surveillance [1]. The HDP and AMS liners (Kyocera, Kyoto, Japan) are polyethylene liners. The implants used for each patient and complications are shown in Table 1.

**References**

1. Kawano S, Sonohata M, Kitajima M, Mawatari M. Highly Cross-linked polyethylene liner dissociation from a cement-less modular acetabular shell: two case reports. Open Orthop J. 2016;10:732-40.

Table 1. Implants used for each patient and complications

| Sex | Age | Leg | Follow-up  Period, months | Acetabular shell | Liner | Ball | Femoral component | Complication |
| --- | --- | --- | --- | --- | --- | --- | --- | --- |
| F | 43 | R | 130 | AMS-HA | AMS | Zirconia | PerFix-HA |  |
|  |  | L | 129 | AMS-HA | AMS | Zirconia | PerFix-HA | Transfusion |
| F | 72 | R | 72 | AMS-HA | ABS | Alumina | PerFix-HA | Liner dissociation |
| F | 53 | R | 187 | AMS-HA | HDP | Zirconia | PerFix-HA | Cup loosening |
|  |  | L | 193 | AMS-HA | HDP | Zirconia | PerFix-HA |  |
| F | 62 | R | 68 | AMS-HA | AMS | Zirconia | PerFix-HA |  |
| F | 61 | R | 61 | AMS-HA | AMS | Zirconia | PerFix-HA | Transfusion |
| F | 45 | R | 101 | AMS-HA | AMS | Zirconia | PerFix-HA | Transfusion |
|  |  | L | 103 | AMS-HA | AMS | Zirconia | PerFix-HA |  |
| M | 61 | R | 101 | AMS-HA | HDP | Zirconia | PerFix-HA | Dislocation, infection |
| F | 62 | R | 160 | AMS-HA | AMS | Zirconia | PerFix-HA | Stem loosening, postoperative shunt blockage |
| F | 52 | R | 132 | AMS-HA | AMS | Zirconia | PerFix-HA | Cup loosening |
|  |  | L | 120 | AMS-HA | AMS | Zirconia | PerFix-HA |  |
| F | 47 | R | 100 | AMS-HA | HDP | Zirconia | PerFix-HA |  |
| F | 54 | R | 156 | AMS-HA | HDP | Zirconia | PerFix-HA | Osteolysis around acetabular shell and femoral component, Transfusion |
| F | 53 | L | 84 | AMS-HA | ABS | Alumina | PerFix-HA |  |
| F | 71 | R | 90 | AMS-HA | AMS | Zirconia | PerFix-HA | Transfusion |
|  |  | L | 96 | AMS-HA | AMS | Zirconia | PerFix-HA |  |
| F | 45 | L | 122 | AMS-HA | AMS | Zirconia | PerFix-HA |  |
| M | 74 | R | 60 | AMS-HA | AMS | Zirconia | PerFix-HA | Postoperative shunt blockage |
| F | 56 | R | 100 | AMS-HA | AMS | Zirconia | PerFix-HA |  |
| F | 44 | R | 72 | AMS-HA | AMS | Zirconia | PerFix-HA |  |
| M | 65 | R | 84 | AMS-HA | AMS | Zirconia | PerFix-HA |  |
| F | 56 | R | 121 | AMS-HA | AMS | Zirconia | PerFix-HA | Transfusion |
| F | 62 | L | 216 | AMS-HA | HDP | Zirconia | PerFix-HA | Periprosthetic fracture |
| F | 55 | L | 60 | AMS-HA | AMS | Zirconia | PerFix-HA | Dislocation, transfusion |
| M | 61 | L | 98 | AMS-HA | AMS | Zirconia | PerFix-HA | Transfusion |
| F | 59 | R | 90 | AMS-HA | AMS | Zirconia | PerFix-HA |  |
|  |  | L | 93 | AMS-HA | AMS | Zirconia | PerFix-HA |  |
| F | 44 | R | 60 | AMS-HA | AMS | Zirconia | PerFix-HA |  |

F, female; L, left; M, male; R, right.
