# Supplementary material for: Hydroxyapatite-coated cementless total hip arthroplasty for patients undergoing dialysis: a study of 30 hips with a minimum follow-up period of 5 years
Source: BMC Musculoskelet Disord. 2021 Sep 30;22:842. doi: 10.1186/s12891-021-04718-3 (PMC8485528; doi:10.1186/s12891-021-04718-3)
Supplement: Supplementary file 2 — Additional file 2. Details of the control group. [file 12891_2021_4718_MOESM2_ESM.docx]

**Additional file 2**

Table 1. Characteristics of the control group

| Hips/patients, n | 26/26 |
| --- | --- |
| Sex, male and female | 3 (11.5) and 23 (88.5) |
| Age at primary THA, years | 56.2 ± 10.0 (42-79) |
| Height, cm | 150.0 ± 8.9 (127.1-157.6) |
| Weight, kg | 44.9 ± 5.4 (32.8-56.5) |
| Body mass index, kg/m^2^ | 20.0 ± 2.1 (16.3-24.7) |
| Follow-up period after primary THA, months | 104.8 ± 33.2 (60-168) |
| Preoperative diagnosis for primary THA |  |
| Osteoarthritis | 22 (84.6) |
| Osteonecrosis | 4 (15.4) |

Data are presented as n (%) or mean ± standard deviation (range). THA, total hip arthroplasty.

Table 2. Clinical results and complications of the control group

|  |  | p-value |
| --- | --- | --- |
| Operative time, min | 38.2 ± 9.0 (25-55) |  |
| Intraoperative blood loss, g | 243.6 ± 145.1 (50-550) |  |
| Postoperative blood loss, g | 438.5 ± 238.0 (120-990) |  |
|  |  |  |
| Harris hip scores |  |  |
| Total before THA | 39.1 ± 12.0 (18-66) | <0.0001 |
| Total at final follow-up | 87.4 ± 9.2 (62-95) |  |
| Pain before THA | 11.5 ± 7.3 (0-30) | <0.0001 |
| Pain at final follow-up | 41.2 ± 5.3 (30-44) |  |
| Function before THA | 24.3 ± 7.8 (6-42) | <0.0001 |
| Function at final follow-up | 42.3 ± 6.8 (18-47) |  |
| Deformity before THA | 0.2 ± 0.6 (0-2) | 1.000 |
| Deformity at final follow-up | 0.2 ± 0.6 (0-2) |  |
| ROM before THA | 3.1 ± 1.1 (1-5) | 0.004 |
| ROM at final follow-up | 3.8 ± 0.5 (3-5) |  |
|  |  |  |
| Autogenous bone graft | 6 (23.1) |  |
|  |  |  |
| Complications |  |  |
| Transfusion | 2 (8.7) |  |

Data are presented as the mean ± standard deviation (range) or number of hips (%).THA, total hip arthroplasty; ROM, range of motion.

Table 3. Radiographic evaluation of the control group

| Acetabular side |  |
| --- | --- |
| Fixation status at final follow-up | Bone ingrowth fixation (24) |
| Femoral side |  |
| Dorr cortical bone classification before primary THA | A (6), B (18), C (2) |
| Stress shielding at final follow-up | 1 (9), 2 (2), 3 (5), 4 (3) |
| Fixation status at final follow-up | Bone ingrowth fixation (24) |

Numbers in parentheses indicate the number of hips in each category. THA, total hip arthroplasty.
